# Antimicrobial and Fermentation Potential of *Himanthalia elongata* in Food Applications

**DOI:** 10.3390/microorganisms8020248

**Published:** 2020-02-13

**Authors:** Francesco Martelli, Claudia Favari, Pedro Mena, Stefano Guazzetti, Annalisa Ricci, Daniele Del Rio, Camilla Lazzi, Erasmo Neviani, Valentina Bernini

**Affiliations:** 1 Department of Food and Drug, University of Parma, Parco Area delle Scienze 49/A, 43124 Parma, Italy; francesco.martelli@studenti.unipr.it (F.M.); claudia.favari@studenti.unipr.it (C.F.); pedromiguel.menaparreno@unipr.it (P.M.); camilla.lazzi@unipr.it (C.L.); erasmo.neviani@unipr.it (E.N.); valentina.bernini@unipr.it (V.B.); 2 Human Nutrition Unit, Department of Veterinary Science, University of Parma, Via Volturno, 39, 43125 Parma, Italy; daniele.delrio@unipr.it; 3 AUSL Reggio Emilia, Via Giovanni Amendola 2, 42122 Reggio Emilia, Italy; stefano.guazzetti@ausl.re.it; 4 School of Advanced Studies on Food and Nutrition, University of Parma, 43121 Parma, Italy

**Keywords:** seaweeds, fermentation, *Himanthalia elongata*, antimicrobial activity, food safety, foodborne pathogens, phlorotannins

## Abstract

*Himanthalia elongata* is a brown oceanic seaweed rich in bioactive compounds. It could play an important role in food production because of its antimicrobial and antioxidant properties. Three strains belonging to the *Lactobacillus casei* group (*Lactobacillus casei*, *Lactobacillus paracasei*, and *Lactobacillus rhamnosus*) and a *Bacillus subtilis* strain were used for the solid-state fermentation of commercial seaweeds, and bacterial growth was monitored using the plate count method. High-pressure processing (HPP) was also employed (6000 bar, 5 min, 5 °C) before extraction. The antimicrobial activity of the extracts was tested in terms of the main food pathogenic bacteria (*Salmonella* spp., *Listeria monocytogenes*, *Escherichia coli*, *Staphylococcus aureus*, and *Bacillus cereus*), and the phenolic content was estimated using the Folin–Ciocalteau method. In addition, targeted UHPLC-MS^2^ methods were used to unravel the profile of phlorotannins. *H. elongata* allowed the growth of the *L. casei* group strains and *B. subtilis*, showing the fermentability of this substrate. Significant antimicrobial activity toward *L. monocytogenes* was observed in the extracts obtained from unfermented samples, but neither fermentation nor HPP enhanced the natural antimicrobial activity of this seaweed species. The content in the phenolic compounds decreased because of the fermentation process, and the amount of phenolics in both the unfermented and fermented *H. elongata* extracts was very low. Despite phlorotannins being related to the natural antimicrobial activity of this brown seaweed, these results did not support this association. Even if fermentation and HPP were not proven to be effective tools for enhancing the useful compounds of *H. elongata*, the seaweed was shown to be a suitable substrate for *L. casei* group strains as well as for *B. subtilis* growth, and its extracts exhibited antimicrobial activity toward foodborne pathogens.

## 1. Introduction

Over the centuries, various preservation techniques have been developed to increase food safety and avoid spoilage during storage and distribution. In recent years, consumers, concerned about the potential side effects of synthetic antimicrobials, have started requesting food that contains preservatives of natural origin [1]. 

This consumer choice has garnered interest in compounds extracted from natural sources, which are increasingly investigated for their antimicrobial activity. Among them, algae represent an interesting raw material, as they are rich in bioactive compounds, and macroalgae have already been proven to have a broad range of applications as antibacterials [2,3,4,5]. Different studies have attributed the antimicrobial and antioxidant capacity of seaweed extracts to the presence of bioactive compounds such as phlorotannins, flavonoids, steroids, and sulfated polysaccharides [6,7,8,9]. These secondary metabolites allow for the synthesizing organism to have a strong defense against pathogens and survive in stressful conditions. Moreover, many of these compounds have been proven to be able to inhibit bacterial growth [10]. Some plant peptides might also exert antimicrobial activity, and can be divided into two groups: (i) endogenous peptides, which are already present in the organism, and (ii) peptides generated by enzymatic hydrolysis and/or fermentation [11]. Moreover, antimicrobial compounds such as organic acids can be produced through fermentation [12]. 

Seaweed fermentation is poorly reported in the literature, although lactic acid bacteria (LAB) and yeast have been employed, after agar–agar extraction, in red seaweed waste fermentation in order to produce fertilizers [13]. The fermentation of *Undaria pinnatifida* (brown seaweed) has been reported as an alternative feed for aquaculture [14], whereas, within the framework of human consumption, a fermented beverage from *Gracilaria fisheri* has been developed [15].

The fermentation of *Himanthalia elongata*, a brown seaweed belonging to the order of *Fucales* (which grows spontaneously along the coasts of the Atlantic Ocean), has been attempted without success, as neither heat-processed nor raw seaweed were able to support the growth of *Lactobacillus plantarum* [16]. However, its antimicrobial activity toward *Escherichia coli* and *Staphylococcus aureus* has been demonstrated [17]. Eom and colleagues have attributed the strong antimicrobial activity of marine brown algae to phlorotannins [18], a subclass of phenolic compounds originating from the polymerization of phloroglucinol (1,3,5-trihydroxybenzene), or to sulfated polysaccharides such as fucoidans. High concentrations of fucose and sulfates, as well as their particular arrangement in brown algal fibers, are probably responsible for this resistance to bacterial fermentation [19]. 

High pressure improves the extraction of active compounds by disrupting tissues, cell walls, membranes, and organelles, consequently increasing the mass contact of the solvents with the samples. This strategy could represent an opportunity to obtain higher extraction yields with no deleterious effects on the activity and structure of potentially bioactive components [20,21,22]. 

Despite the evidence for the antimicrobial activity of algae and the role of fermentation in the production of bioactive compounds, screening different bacterial species for fermentation and thus producing antimicrobial compounds from fermented seaweed has never been tested. For this reason, the aim of the present study was to evaluate (i) the capacity of the *Lactobacillus casei* group of bacteria and *Bacillus subtilis* to ferment *H. elongata*; (ii) the antimicrobial activity of the *H. elongata* extract in foodborne pathogenic bacteria; and ii) the effect of biological (fermentation) and technological (high-pressure processing (HPP)) processes on the antimicrobial activity and phenolic composition of *H. elongata*.

## 2. Materials and Methods

### 2.1. Sample Preparation

*H. elongata* samples were purchased (dried) from ALGAMAR (Pontevedra, Spain) (*HE* I) and Nuova Terra (Prato, Italy) (*HE* II) in order to evaluate variability within the species. They were ground with Oster 890-48H mixer (Recampro, Spain) and maintained at room temperature in darkness until use.

### 2.2. Standards and Reagents

All chemicals and solvents, which were purchased from Sigma–Aldrich Co. (St. Louis, MO, USA), were of analytical grade. Ultrapure water from a MilliQ system (Millipore, Bedford, MA, USA) was used throughout the experiment.

### 2.3. Bacterial Strains Used for Fermentation

Four bacterial strains isolated from food matrices and belonging to different species were used to ferment *HE* I: *Lactobacillus casei* (2240) and *Lactobacillus rhamnosus* (1473) (isolated from Parmigiano Reggiano cheese), *Lactobacillus paracasei* (4186) (isolated from Pecorino Toscano cheese), and *Bacillus subtilis* (5002) (isolated from rice). The strains, which belonged to the collection of the Department of Food and Drugs of the University of Parma, were maintained at −80 °C in de Man, Rogosa, and Sharpe (MRS) (Oxoid, Basingstoke, UK) (for the LAB) and in Nutrient Broth (Oxoid) (NB) (for *B. subtilis*) with 12.5% glycerol (*v*/*v*) added.

### 2.4. Set-Up of Fermentation Conditions and Monitoring

Before fermentation, the frozen cultures were revitalized twice in MRS broth (Oxoid) (inoculum of 3% *v*/*v*) incubated overnight at 37 °C under suspended conditions (for the LAB) and in NB incubated overnight at 30 °C under shaking (for *B. subtilis*). Afterwards, LAB and *B. subtilis* were inoculated (3% *v*/*v*) in MRS and NB and incubated at specific temperatures for each species (for 16 h) in order to obtain a concentration of 9 log cfu/mL. The grown cell cultures were collected by centrifugation (12,857× *g* for 10 min at 4 °C), washed twice in Ringer solution (Oxoid, Milan, Italy), and resuspended in sterile bidistilled water. *HE* I was rehydrated with 75% of water and then inoculated individually with each bacterial suspension in order to obtain a final concentration of 7 log cfu/mL. The microbial concentration was evaluated just after the inoculum (*T*0), after 24 h (*T*1) and 72 h (*T*2) of fermentation at the optimal temperature for each strain. Ten-fold serial dilutions in Ringer (Oxoid) were plated on MRS agar or nutrient agar (NA) (for LAB and *B. subtilis*, respectively) and then incubated for 72 h in aerobic conditions at the optimal temperature for each strain. Fermentation was carried out in duplicate, and for each sample, time analyses were performed in duplicate. Average values ± standard deviations are reported. After the process, fermented seaweeds were lyophilized.

### 2.5. High-Pressure Processing (HPP)

High-pressure treatments were performed using equipment from HPP Italia (Traversetolo, Parma, Italy) on *HE* I. Seaweeds were first rehydrated for 30 min with 75% water at room temperature, submitted to a vacuum in bags, and then placed into containers in a hyperbaric chamber that was then filled with cold water for treatment. The process was carried out at 6000 bars for 5 min at 5 °C. After the treatment, the product was lyophilized. Samples were treated and analyzed in duplicate. Average values ± standard deviations are reported.

### 2.6. Extraction Process 

In order to extract molecules with potential antimicrobial activity, such as polyphenols, small peptides, and acids, an extraction process from *HE* I (unfermented, fermented, and HPP-treated) and *HE* II (unfermented) was carried out. In particular, 100 mL of ethanol/water (70:30 *v*/*v*) acidified with 1% formic acid (CH_2_O_2_) was added to 10 g of lyophilized sample. A double extraction was carried out, alternating two shaking cycles and one sonication cycle in an ultrasonic bath, with each lasting 15 min. An HS 501 digital shaker (IKA) (Staufen, Germany) was used for the shaking cycle (200 rpm), while the sonication was carried out by means of an Ultrasonic Cleaner sonicator (VWR, United States). The sample was then centrifuged (Eppendorf 5800 Centrifuge, Model 5810R, Hamburg, Germany) at 12,857× *g* for 10 min at 10 °C. The solution was filtered with filter paper to recover the solid part so as to proceed to the second extraction. The two extracts obtained were combined and concentrated with a rotary evaporator Strike 300 (Steroglass, Italy) at 4× *g* at a bath temperature of 40 °C until they were fully dried. The concentrated extract was then suspended using sterile water to recover the soluble part and stored at −80 °C until use. To test stability at a high temperature, the *HE* I extract underwent a treatment at 121 °C for 15 min using an autoclave.

### 2.7. Pathogenic Strains

The antimicrobial activity of the extracts obtained from unfermented, fermented, and HPP-treated *HE* I and unfermented *HE* II was tested in terms of 14 pathogenic strains belonging to *Salmonella* spp. (S1: *S. enterica* ATCC 14028; S2: *S. enterica* serotype *Rissen*; and S3: *Salmonella* spp. suini), *Listeria monocytogenes* (L1: LM30; L2: LMG 21264; and L3: LMG 13305), *Escherichia coli* (E1: DSM 9025; E2: DSM 10973; and E3: POM 1048), *Staphylococcus aureus* (A1: NCTC 9393; A2: ATCC 6538; and A3: ATCC 19095), and *Bacillus cereus* (C1: 31; C2: 33). These strains were part of the collection of the Department of Food and Drugs (University of Parma, Italy) and were part of international collections, including the National Collection of Type Cultures (NCTC), the Belgian Coordinated Collection of Microorganisms (LMG), the American Type Culture Collection (ATCC), and the Deustsche Sammlung von Mikroorganismen (DSM). They were stored at −80 °C in Tryptic Soy Broth (TSB) (Oxoid) supplemented with 12.5% glycerol (*v*/*v*). Before use, bacteria were revitalized twice by inoculum (3% *v*/*v*) in TSB with 0.6% yeast extract followed by incubation for 16 h at 37 °C in aerobic conditions. 

### 2.8. Evaluation of Antimicrobial Activity In Vitro

An evaluation of the antimicrobial activity of the extracts was carried out using an agar well diffusion assay [23] with few modifications. The pathogenic strains were diluted to a concentration of 8 log cfu/mL and seeded on Tryptone Soy Agar (TSA) (Oxoid) by means of sterile swabs. Then, using sterile tips, wells with a diameter of 7 mm were created in the agar and filled with 30 μL of each extract. Plates were incubated at 37 °C in aerobic conditions, and the antimicrobial activity was evaluated by measuring the total inhibition zone (mm) observable after 24, 48, and 120 h of incubation. Analyses were performed in triplicate, and average values ± standard deviations are reported. Water was used as a negative control.

### 2.9. Total Phenolic Content

The total phenolic content (TPC) of the extracts obtained from the unfermented, fermented, and HPP-treated *HE* I and the unfermented *HE* II was determined using the Folin–Ciocalteau method, as outlined by Medina-Remón and colleagues [24] (with slight modifications) [25]. Briefly, 15 μL of diluted sample was mixed with 170 μL of double-distilled water in 96-well microplates (Sarstedt AG & Co., Nümbrecht, Germany), and then 12 μL of Folin–Ciocalteau’s reagent and 30 μL of sodium carbonate (200 g/L) were added. The mixtures were kept at room temperature in darkness for 1 h. After the reaction period, 73 μL of double-distilled water was added, and absorbance was recorded at 765 nm on a Sunrise™ microplate reader (Tecan, Grödig, Austria). Sample quantification was performed using gallic acid as a standard, and the results are expressed as mg of gallic acid equivalents (GAEs) per gram of dry weight (DW). Analyses were performed in triplicate, and average values ± standard deviations are reported.

### 2.10. UHPLC-ESI-MS^2^ Analysis

The extracts obtained from the unfermented, fermented, and HPP-treated *HE* I and the unfermented *HE* II were analyzed using ultrahigh-performance liquid chromatography (UHPLC) coupled with mass spectrometry (MS) to investigate the presence of phlorotannins. To prepare for the analysis, extracts were centrifuged at 15,294× *g* for 10 min, diluted in 0.1% formic acid in water (1:2, *v*/*v*), vortexed, centrifuged once again at 10,625× *g* for 10 min at 4 °C, and finally filtered (0.45-μm nylon filter). An Accela UHPLC 1250 apparatus equipped with a linear ion trap MS (LIT-MS) (LTQ XL, Thermo Fisher Scientific Inc., San José, CA, USA) was used. The separation of the compounds was carried out by means of an Acquity UPLC HSS T3 column (100 × 2.1 mm, 1.8-μm particle size, Waters, Milford, MA, USA). For the UHPLC, mobile phase was pumped at a flow rate of 0.3 mL/min, and it consisted of a mixture of acidified acetonitrile and 0.1% formic acid (solvent A) and 0.1% formic acid in water (solvent B). Following 1.50 min of 1% solvent A in B, the proportion of A was linearly increased to 38.3% over 7.10 min, reaching 90% solvent A at 10 min, followed by 3 min of 90% solvent A and then 4 min at the initial conditions to re-equilibrate the column. The injection volume was 5 μL, and the column was put on a thermostat at 40 °C. The MS worked in negative ionization mode with the capillary temperature set at 275 °C and the source at 250 °C. The sheath gas (N_2_) flow was 40 units, while the auxiliary gas (N_2_) flow was 10 units. The source voltage was 4 kV, while the capillary voltage and the tube lens voltage were −50 V and −142.75 V, respectively. Targeted MS^2^ analyses were carried out to identify the phlorotannins using the fragmentation of specific molecular ions (*m*/*z*). Identification was performed through a comparison to spectral data that have been reported in literature.

### 2.11. Statistical Analyses

The analyses were conducted separately for *Salmonella* spp., *E. coli*, *S. aureus*, *L. monocytogenes*, and *B. cereus*, and for each, two different analyses were performed: one for the extract obtained from nonfermented (time of fermentation = 0) seaweed and one for the extracts obtained after fermentation. For both, the effects of different parameters on the inhibition zone were studied by means of linear mixed effects models (LMMs). The radius of the inhibition zone was measured at 24, 48, and 120 h after inoculum. In the statistical analyses of the nonfermented extracts ((i) *HE* I and *HE* II, (ii) *HE* I that was HPP-treated, and (iii) *HE* I that was sterilized), the effect of the incubation time, the effect on the pathogenic bacterial strains, and the interaction between the time of incubation and the kind of extract were considered to be “fixed effects”, while the effects of the plate and the section nested within the plate were considered to be random effects. In the analyses of the extracts obtained after fermentation, the time of fermentation (24 and 72 h), the effect of the different strains used for fermentation (*B. subtilis* taken as a reference), the effect on the pathogenic strains, the effect of the incubation time (24, 48, and 120 h), and the interaction between the time of incubation and the type of extract were considered to be fixed effects. The plate, the section nested within the plate, and the batch of fermentation were considered to be possible sources of nonindependence. All of the analyses were conducted with R [26], and the LMM model analyses were conducted using the “lme4” package [27]. For analyses related to the extracts’ phenolic content, the SPSS statistical package (version 25, SPSS, Inc., Chicago, IL, USA.) was used. One-way analysis of variance (ANOVA) with Duncan post hoc tests, as well as *t*-tests, were carried out.

## 3. Results and Discussion

### 3.1. Himanthalia elongata Fermentation

**Figure 1:**
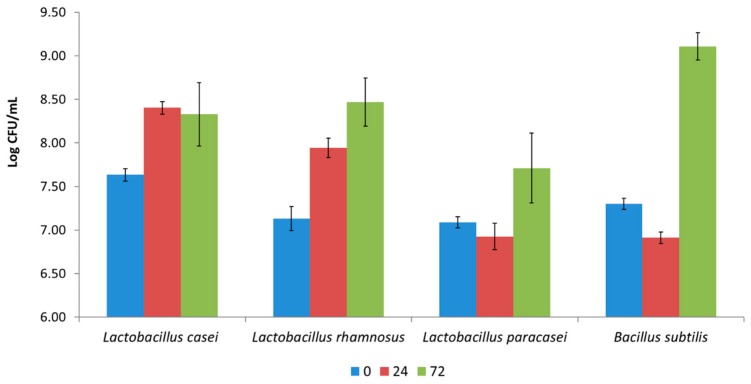
The growth ability of the *Lactobacillus casei* group and the *Bacillus subtilis* strains (log cfu/mL) in *Himanthalia elongata* after 24 and 72 h of fermentation at optimal temperatures.

All strains showed different but generally good growth capacity on *H. elongata* (Figure 1). Contrary to Gupta et al. [16], who observed that neither raw nor thermally processed *H. elongata* were able to support the growth of *L. plantarum*, in the present work the strains used for fermentation (*L. casei* 2240, *L. paracasei* 4186, *L. rhamnosus* 1473, and *B. subtilis* 5002) demonstrated the capacity to grow in this matrix, opening new avenues for novel fermented foods based on algae. To the best of our knowledge, the species tested in the present study have never been employed for *H. elongata* fermentation. However, *Weissella* spp., *Lactobacillus* spp., *Leuconostoc* spp., *Streptococcus* spp., and *L. rhamnosus* have been employed for the fermentation of different algal species (showing good growth ability) [28,29,30].

### 3.2. Antimicrobial Activity toward Foodborne Pathogens

**Figure 2:**
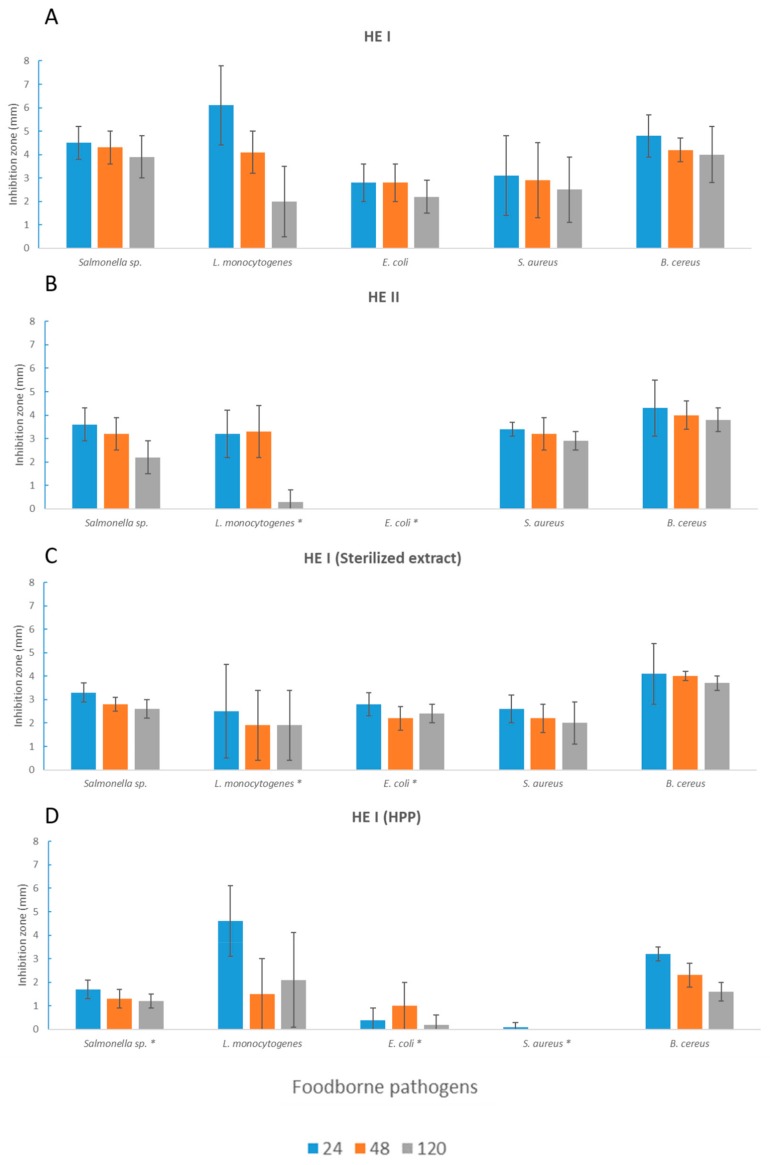
Inhibition radius (mm) of extracts obtained from unfermented seaweed in terms of foodborne pathogens (**A**: *HE* I, **B**: *HE* II, **C**: sterilized extract obtained from *HE* I, **D**: *HE* I treated with HPP). Measurements were taken after 24, 48, and 120 h. Values ± standard deviations are reported. Here, * expresses the significance (*p* < 0.05) of the antimicrobial activity of *HE* I and of the other extracts (*HE* II, *HE* I (sterilized extract), and *HE* I (high-pressure-processed (HPP))) for each time point (24, 48, and 120 h).

Currently, the antimicrobial potential of seaweed toward the main foodborne pathogens is one of the most stimulating fields of research in the area of “marine vegetables”. It has been demonstrated that brown algae extracts are the most effective against foodborne pathogens [31]. Several seaweed species have been studied for their rich content in bioactive compounds, and their antimicrobial activity has been well known for many years [32]. Each class of seaweed (Phaeophyceae, Rhodophyta, and Chlorophyta), because of their different compositions, has a different degree of antimicrobial activity and different target microorganisms [33]. *Ecklonia cava*, a brown seaweed, has been tested on *L. monocytogenes*, and good antimicrobial activity was found [34]. Several *Sargassum* species have been found to be strong antimicrobial agents, mainly against Gram-positive bacteria but also toward *Salmonella* spp. [33]. Seaweeds offer opportunities for obtaining new types of bioactive compounds that could be used by the food industry as preservatives; however, the mechanisms of inhibition of seaweed extracts are not always clear. The antimicrobial activity of *H. elongata* compounds has been previously studied [17,22,35], but the effects of fermentation and of HPP on these algae have never been considered. In the present work, the inhibitory activity of algae extracts against the main food pathogenic bacteria was evaluated. As a first observation, the extracts obtained from the nonfermented seaweeds inhibited the target pathogens in a different way (Figure 2). 

*HE* I and *HE* II were provided from different suppliers in order to evaluate the possible variability within the same species. Indeed, the two extracts showed significantly different behavior against *L. monocytogenes* (*p* < 0.05) and *E. coli* (*p* < 0.001) (Figure 2). However, most of the pathogenic species tested were affected by both extracts. Antimicrobial activity toward different pathogens could be related to the wide number of compounds observed in seaweeds (polysaccharides, polyunsaturated fatty acids, phlorotannins, other phenolic compounds, carotenoids, etc.) [31]. Nevertheless, many natural factors, such as environmental conditions (light, temperature, and salinity), life stage (the age of seaweed), geographical location, and the seasonality of growth and harvesting, can be influential as well [36,37]. 

Since *HE* I had a better antimicrobial performance than did *HE* II, it was subjected to different treatments, such as a high temperature, fermentation, and HPP, in order to check the effects on antimicrobial activity.

The *HE* I extract showed good heat resistance, as the thermal treatment induced a significant reduction in antimicrobial activity (but only toward *L. monocytogenes* (*p* < 0.05)) (Figure 2). The resistance of the extract to high temperatures could be useful for applications in thermally processed products to prevent the occurrence of postprocessing contamination. In order to study the possibility of enhancing seaweed antimicrobial activity, a high-pressure treatment, considered to be a promising strategy for the extraction of bioactive ingredients from plant material [21], was performed. However, the antimicrobial activity did not increase, but rather, efficacy against *Salmonella* spp. (*p* < 0.001), *E. coli* (*p* < 0.001), and *S. aureus* (*p* < 0.05) was reduced after HPP treatment (Figure 2). 

All of the extracts from nonfermented samples, regardless of the seaweed and the treatment, lost inhibitory activity toward all of the tested microorganisms after 120 h of incubation at the optimum growth temperature, and this was already significant after 48 h for *L. monocytogenes* (*p* = 0.001) (Figure 2). 

**Figure 3:**
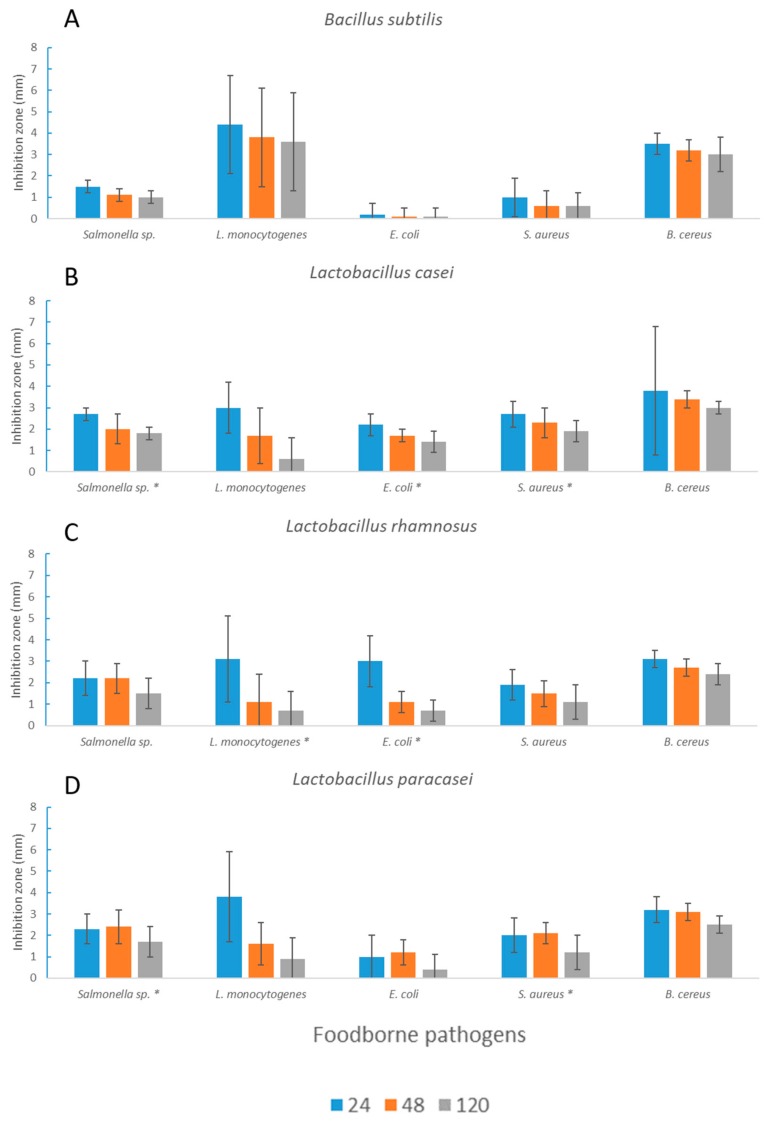
Inhibition radius (mm) of fermented *HE* I extracts after 24 h of fermentation. Measurements were taken after 24, 48, and 120 h. Average values ± standard deviations are reported. Here, * expresses the significance (*p* < 0.05) of *H. elongata* fermented with *B. subtilis*, A, and the same seaweed fermented with the *L. casei* group (*L. casei*, B, *L. rhamnosus*, C, and *L. paracasei*, D) of strains.

**Figure 4:**
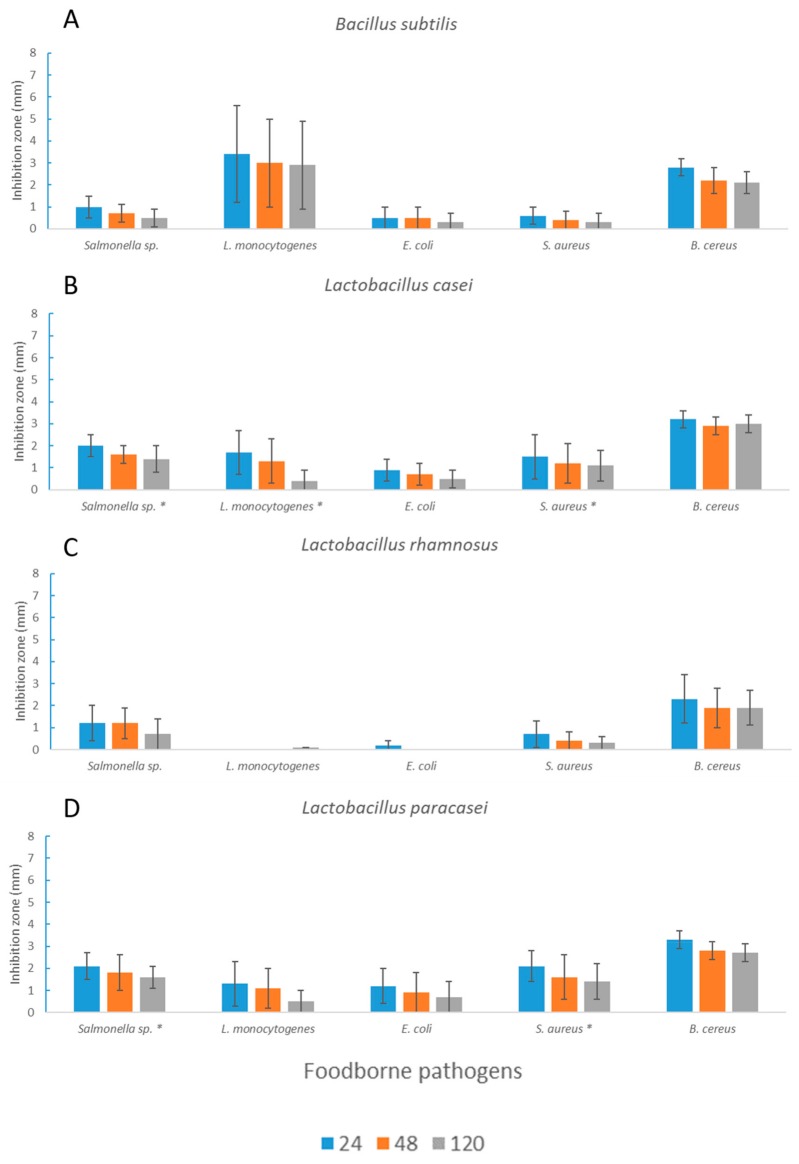
Inhibition radius (mm) of *HE* I extracts after 72 h of fermentation. Measurements were taken after 24, 48, and 120 h. Average values ± standard deviations are reported. Here, * expresses the significance (*p* < 0.05) of *H. elongata* fermented with *B. subtilis*, A, and the same seaweed fermented with the *L. casei* group (*L. casei*, B, *L. rhamnosus*, C, and *L. paracasei*, D) of strains.

*HE* I was also tested after *L. casei*, *L. rhamnosus*, and *L. paracasei* fermentation to evaluate the contribution of microorganisms and their metabolic activity (Figure 3 and Figure 4). 

Extracts after LAB fermentation gave rise to stronger antimicrobial activity compared to those obtained after fermentation with *B. subtilis* (with the exception of *L. monocytogenes*). The extracts obtained after *L. casei* and *L. paracasei* fermentation showed significantly higher activity toward *Salmonella* spp. and *S. aureus*, whereas in the case of *L. rhamnosus*, significantly lower antimicrobial activity toward *L. monocytogenes* (*p* < 0.05) but significantly higher activity toward *E. coli* (*p* < 0.05) could be observed. Overall, the extracts obtained after seaweed fermentation significantly lost their activity toward all of the tested pathogenic bacteria (except for *E. coli*) over time (Figure 3 and Figure 4). 

**Figure 5:**
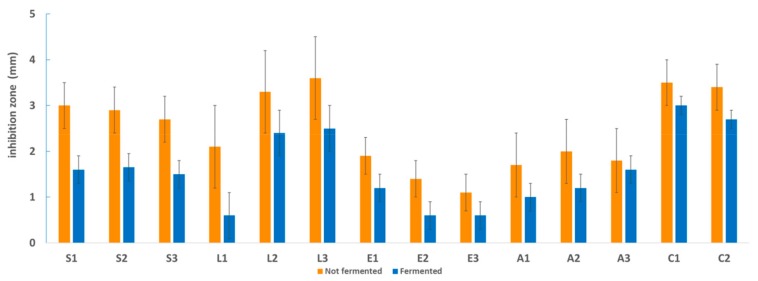
Antimicrobial activity of extracts from nonfermented and fermented samples (average values) toward each strain of foodborne pathogenic bacteria: *Salmonella* spp. (S1: *S. enterica* ATCC 14028; S2: *S. enterica* serotype *Rissen*; and S3: *Salmonella* spp. suini), *Listeria monocytogenes* (L1: LM30; L2: LMG 21264; and L3: LMG 13305), *Escherichia coli* (E1: DSM 9025; E2: DSM 10973; and E3: POM 1048), *Staphylococcus aureus* (A1: NCTC 9393; A2: ATCC 6538; and A3: ATCC 19095), and *Bacillus cereus* (C1: 31; C2: 33).

A comparison of the antimicrobial activity (average values) of extracts derived from fermented s (or not) seaweeds is reported in Figure 5. Fermentation did not enhance the natural antimicrobial activity of this brown seaweed. Recent papers regarding plant fermentation have shown a significant enhancement of antimicrobial activity toward pathogens [11,38,39], but this was not confirmed in the case of *H. elongata* in the present work. Possibly, some bacterial catabolic activity broke down the compounds originally exerting the antimicrobial activity in the raw extracts, resulting in lower efficacy. 

### 3.3. Total Phenolic Content

The TPC was similar for the *HE* I and *HE* II extracts obtained from the unfermented samples, with values of 2.94 ± 0.28 mg GAEs/g DW and 3.22 ± 0.21 mg GAEs/g DW, respectively. The results from the Folin–Ciocalteau assay showed that the TPC was significantly higher for extracts derived from nonfermented samples compared to fermented ones. Quantitatively, fermentation caused a 10-fold decrease in TPC value, while the time of fermentation (24 and 72 h) did not affect the TPC Table 1. After 24 h of fermentation, *L. casei* led to a smaller reduction in the TPC compared to the other microorganisms, while *L. casei* and *L. paracasei* were responsible for a greater TPC reduction after 72 h of fermentation Table 1. Regarding HPP, the extract derived from the HPP-treated seaweed had a TPC that was slightly lower (2.72 ± 0.03 mg GAEs/g DW) than that of the corresponding untreated extract (2.94 ± 0.28 mg GAEs/g DW), but the difference was not statistically significant. This might indicate that HPP does not influence the seaweed extract TPC. In general, it should be noted that the TPC of all of these seaweed extracts was quite low, in line with the literature [22,40,41]. A high variability in the TPC of seaweeds, in particular that of *H. elongata*, has been previously reported, due to multiple factors such as collection season and geographical region [9,40].

### 3.4. Phlorotannin Identification by UHPLC-MS^2^ Analysis

The UHPLC-MS^2^ targeted analyses we used allowed for the tentative identification of a total of 20 phlorotannins (Table 2). The compounds were tentatively identified based on an interpretation of their mass spectral behavior, which was obtained from MS^2^ experiments and through a comparison to the literature [42,43,44]. The identified phlorotannins exhibited distinct molecular weights (370–870 Da) and degrees of polymerization (3–7 phloroglucinol units). Interestingly, all of the compounds were identified in *HE* II (Table 2), while none were found in *HE* I. This could have been due to many factors that occur during seaweed cultivation and growth, but quite possibly it was due to the lesser exposure of *HE* I to sources of stress, such as microbial infections or UV radiation [42,45]. A quantification was not carried out due to the lack of proper commercially available standards for this particular group of hydrolysable tannins.

## 4. Conclusions

Seaweeds are an underestimated and suitable source of food and food ingredients. Their richness in terms of bioactive compounds makes them a possible source of food preservatives and other useful molecules. The results highlighted that *H. elongata* extracts were more efficient against *L. monocytogenes* and *B. cereus*. The extracts obtained from *H. elongata* showed antimicrobial activity against various food pathogenic bacteria. A great difference was observed between *HE* I and *HE* II: despite being the same species, a lot of variability, probably due to environmental factors or to treatments suffered before commercialization, emerged. The tested treatments (sterilization, HPP, and fermentation) negatively affected the antimicrobial activity of *H. elongata*. In conclusion, (i) *H. elongata* was demonstrated to be a suitable substrate for the *L. casei* group of bacteria and *B. subtilis* growth; (ii) its extract exhibited antimicrobial activity toward foodborne pathogens; (iii) fermentation was not an appropriate technology for obtaining innovative antimicrobial compounds from *H. elongate*; (iv) HPP, which is often used as a tool to improve the extraction of bioactive compounds from plant matrixes, did not enhance the natural antimicrobial activity and phenolic content of this seaweed; and (v) the content of the phenolic compounds decreased as a consequence of the fermentation process. Further studies are required to better understand the compounds behind the antimicrobial activity of *H. elongata* extracts.

## Figures and Tables

**Table 1:** Total phenolic content (TPC) of *H. elongata* extracts obtained after fermentation.

		*HE* I	24 h	72 h
TPC (mg GAEs/g DW)	*Lactobacillus casei*	2.94 ± 0.28 ^a^	0.27 ± 0.01 ^b,A^	0.21 ± 0.02 ^b,B^
	*Lactobacillus paracasei*	2.94 ± 0.28 ^a^	0.20 ± 0.00 ^b,B^	0.20 ± 0.02 ^b,B^
	*Lactobacillus rhamnosus*	2.94 ± 0.28 ^a^	0.22 ± 0.01 ^b,B^	0.32 ± 0.08 ^b,A^
	*Bacillus subtilis*	2.94 ± 0.28 ^a^	0.20 ± 0.02 ^b,B^	0.36 ± 0.00 ^b,A^

Values are presented as the mean ± the SD (*n* = 3). TPC is expressed as mg gallic acid equivalents (GAEs)/g dry weight (DW). Means within each row with different letters (a, b) differ significantly (*p* < 0.05), and means within the 24-h and 72-h columns with different letters (A, B) differ significantly (*p* < 0.05).

**Table 2:** Mass spectral characteristics of the tentatively identified phlorotannins in *Himanthalia elongata* (*HE* II).

Compound	RT (min)	[M–H]^–^ (*m*/*z*)	MS^2^ Ion Fragments(*m*/*z*)	Ref.
Trimer	4.56	369	351	a
Trimer	5.16	369	279, 351, 325, 307	a
Trimer	5.34	369	351, 295, 325, 307	a
Trimer	5.65	369	351, 279, 325, 307	a
Trimer	7.00	369	238	a
Trimer	7.14	369	238	a
Trimer	1.50	373	355, 207, 329, 165	b
Trimer (Phlorethol)	5.39	373	231, 355	c
Trimer (Fucophlorethol)	5.75	373	233, 247, 229, 355, 125	c
Tetramer	5.22	497	479, 407, 371	
Tetramer	5.40	497	479, 353, 371, 335	b
Tetramer	5.85	497	235	a
Tetramer	6.36	497	355, 371, 479	a
Tetramer	6.69	497	373, 371, 233, 353, 238, 479	b
Pentamer	5.95	621	603, 339, 337	a,b
Pentamer	6.22	621	603, 339, 357, 337, 229	a,b
Pentamer (Fucol)	7.16	621	495, 371, 497, 477, 229, 603	c
Pentamer (Fuhalol)	8.06	651	509, 465, 413, 607, 339, 582	c
Hexamer	6.51	745	727, 601	a
Heptamer	7.12	869	851, 842, 833	b,c

RT: retention time; [M–H]^−^: molecular ion; fragment ions are listed in order of relative abundance; ^a^ tentatively identified based on the mass spectral data reported by Reference [42]; ^b^ tentatively identified based on the mass spectral data reported by Reference [44]; ^c^ tentatively identified based on the mass spectral data reported by Reference [43].
